# Aerobic Exercise and HFrEF: Impact of Sex and Biological Age

**DOI:** 10.1016/j.jacadv.2025.101658

**Published:** 2025-03-14

**Authors:** Branden L. Nguyen, Zachary S. Clayton

**Affiliations:** Division of Geriatric Medicine, Department of Medicine, University of Colorado Anschutz Medical Campus, Aurora, Colorado, USA

**Keywords:** aging, exercise training, heart failure, sex as a biological variable

**Figure:**
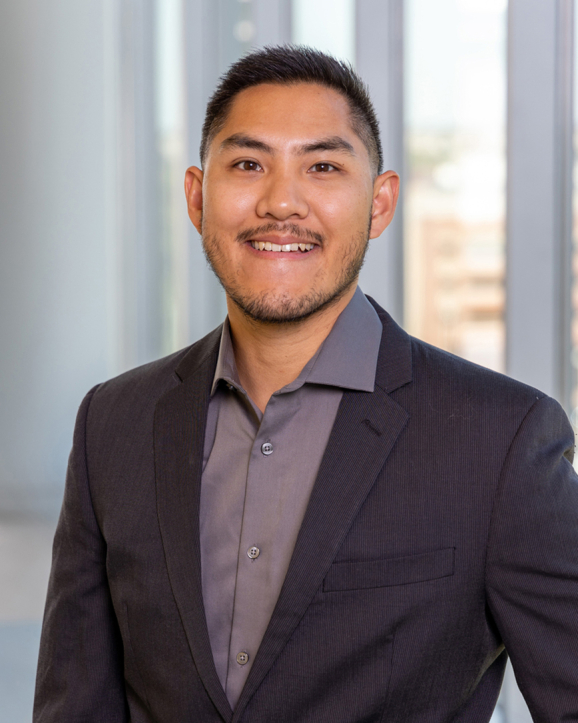

Heart failure (HF) is a pandemic that affects >64 million people.^1^ Recent projections predict the prevalence of HF to increase by 46% from 2012 to 2030, resulting in a significant rise in health care costs.^2^ HF is a clinical syndrome with signs and/or symptoms caused by structural (eg, left ventricular [LV] hypertrophy) and/or functional cardiac abnormalities (eg, reduced LV ejection fraction) and corroborated by elevated circulating concentrations of natriuretic peptide (a biomarker of cardiac dysfunction) and/or objective evidence of pulmonary or systemic congestion.^3^ HF can be further characterized into HF with reduced ejection fraction (HFrEF). HFrEF is defined by LV ejection fraction ≤40% and is associated with progressive LV dilation and adverse LV remodeling.^4^ The prevalence of HFrEF is a major biomedical problem, and the Global Congestive Heart Failure study found that 54% of HF patients had HFrEF.^5^

The global population of older adults is increasing at a remarkable rate, with people at 65 years of age or older expected to rise from 10% in 2022 to 16% in 2050.^6^ A nonmodifiable risk factor for HF is advancing age. For example, HF prevalence was 8.4% in people ≥75 years of age (older adults) compared to 0.7% in people 45 to 54 years of age (middle-age).^7^ In the Biomarkers of Cardiovascular Risk Assessment in Europe study, the risk of developing HF was found to be approximately 38% at 90 years of age.^8^ In addition to chronological age, biological age (BA) is a risk factor for HF. BA can be determined molecularly (eg, telomere length and epigenetics) and/or phenotypically (eg, blood pressure, LV function, and frailty).^9^ BA can be influenced by numerous factors (eg, diet, physical activity, and cancer) that can impact the risk cardiovascular disease (CVD) and HF-related mortality. Indeed, assessment of BA has shown to be a valid and reliable method to predict the risk of all-cause mortality. However, the association of BA and mortality in individuals with HFrEF is unknown. Studies aimed at elucidating BA as a potential predictor of CVD risk and HF-related mortality hold promise for establishing new therapeutic targets.^8^

With the rising older adult population and prevalence of HFrEF, it is biomedically imperative to establish means for mitigating CVD risk and HF. Aerobic exercise training (AET) is a widely effective intervention to improve cardiovascular (CV) health and reduce CV-related mortality in patients with HF.^10^ The HF-ACTION (Heart Failure: A Controlled Trial Investigating Outcomes of Exercise Training) trial has demonstrated that AET in patients with HFrEF improves health outcomes and reduces all-cause mortality compared to standard of care (eg, pharmacological therapies).^11^ Moreover, AET is a well-established intervention for reducing CVD risk in older men and women.^12^ Together, there is strong evidence that AET can improve CV health in both patients with HF and in older adults. However, the effect of AET on BA in individuals with HFrEF is understudied.

In this issue of *JACC: Advances*, Huang et al^13^ sought to determine whether AET in HFrEF patients alters BA, health outcomes, and mortality. This novel study utilized the National Heart, Lung, and Blood Institute’s Biological Specimen and Data Repository Information Coordinating Center and the HF-ACTION database for accessing results, providing one of the largest studies to date evaluating the effects of AET on BA and health outcomes in patients with HFrEF. This study implemented the Kemera-Doubal method (KDM) for analyzing BA—a validated method for predicting age-related health outcomes. They found that greater KDM-BA was associated with a higher risk of all-cause mortality, CV-related mortality, and all-cause hospitalization in the group of patients with HFrEF receiving standard of care. Interestingly, individuals with HFrEF with elevated risk of all-cause mortality who completed the AET intervention had reduced the risk of all-cause mortality compared to standard of care. AET in HFrEF individuals with low risk of all-cause mortality did not differ from the standard-of-care group. These data suggest that AET may be effective in improving BA factors specifically in patients with HFrEF at elevated risk for all-cause mortality (ie, advanced BA).

In comparison to the study by Huang et al, the HF-ACTION trial found that AET did not improve biomarker-based BA. However, a post-hoc analysis of data from the HF-ACTION and REHAB-HF studies identified similar results to Huang et al in that AET-related benefits were more pronounced in patients with elevated risk for all-cause mortality (ie, individuals with an elevated frailty index score). Therefore, these data suggest that the effectiveness of AET to reduce all-cause mortality may only be seen in individuals at higher risk of all-cause mortality. Why the benefits of AET in low-risk all-cause mortality HFrEF individuals were not evident requires further investigation.

The differing responses to AET in HF are complex and require additional studies. A contributing factor that may influence the response to AET is the differing levels of BA as well as sex and gender. There are substantial differences in the CV response to AET with aging between men and women, which may be due to the presence (or lack thereof) of sex steroid hormones.^14^ There is abundant evidence that AET in middle-aged/older men and premenopausal women reduces CVD risk by improving CV function; however, estrogen-deficient postmenopausal women typically do not consistently have the same response.^14^ In the study by Huang et al,^13^ the average age of participants was approximately 59 years of age, with the average distribution of females ranging from 25% to 29%. It is unclear if menopausal state was considered in the study, as the average age for menopause in the United States is approximately 52 years. Therefore, the influence of AET on BA in HFrEF individuals could be influenced by sex and sex steroid hormones.

While Huang et al^13^ demonstrated that the risk of all-cause mortality is reduced with AET in individuals with HFrEF at higher risk of all-cause mortality, it is unclear how the risk of all-cause mortality was determined. The term refers to death from any cause; thus, it is fair to question what specific factors influence it. In contrast, risk factors associated with CVD prevalence have been well defined, allowing for a clearer interpretation of CV-related mortality (ie, cause-specific mortality). Huang et al demonstrate that KDM-BA is a sufficient predictor of all-cause mortality, regardless of chronological age, highlighting the potential of using BA as a means of determining risk factors associated with all-cause mortality.

Nonetheless, the effect of AET in individuals with HFrEF is understudied. Huang et al provide the premise that AET may reduce all-cause mortality and improve patient quality of life in HFrEF individuals with advanced BA. Utilizing AET as a nonpharmacological prophylactic intervention to improve health outcomes and reduce CVD risk holds promise for patients with HFrEF. It is imperative that the field continues to investigate the benefits of AET in individuals with HF. Considering the variability in AET response with aging and HF, future work is warranted to interrogate sex differences and BA factors that may ultimately influence the effect of AET on CV health.

## Funding support and author disclosures

Dr Nguyen is currently funded by NIH R00 HL159241. Dr Clayton is currently funded by NIH T32 AG000279. The authors have reported that they have no relationships relevant to the contents of this paper to disclose.
